# Acute Suspected Stroke Clinical Pathway Use in Tasmanian Emergency Departments: A Retrospective Study

**DOI:** 10.1111/1742-6723.70254

**Published:** 2026-04-07

**Authors:** Innocent Tawanda Mudzingwa, Sarah Jane Prior, Phoebe Griffin, Emma Tavender, Viet Tran

**Affiliations:** ^1^ Tasmanian School of Medicine University of Tasmania Hobart Australia; ^2^ Tasmanian School of Medicine University of Tasmania Burnie Australia; ^3^ Tasmanian School of Medicine University of Tasmania Launceston Australia; ^4^ Emergency Research, Murdoch Children's Research Institute, Departments of Paediatrics and Critical Care University of Melbourne, Australian Catholic University Melbourne Australia

**Keywords:** emergency department, regional EDs, suspected stroke pathway

## Abstract

**Introduction:**

Stroke is a leading cause of disability and healthcare burden in Australia, affecting over 440,000 people. Timely diagnosis and intervention are essential for improving outcomes, and national ED standards emphasise rapid assessment, imaging and treatment. Clinical pathways aim to standardise care and reduce delays, yet regional disparities persist. This study examined suspected acute stroke pathway use in Tasmanian EDs, assessing guideline adherence, waiting times and inter‐hospital variation.

**Methods:**

This retrospective observational study analysed 205 suspected stroke presentations across four public Tasmanian EDs in 2021. Data from digital medical records were evaluated for pathway utilisation, adherence to key clinical elements and time metrics.

**Results:**

The mean patient age was 70.5 years, with 56% male. Suspected stroke pathway use varied significantly between hospitals (27%–62%). In unadjusted analyses, pathway‐managed patients more frequently completed ROSIER and NIHSS assessments (*p* < 0.0025) and were more likely to receive thrombolysis, though causality cannot be inferred. Pathway use was also associated with shorter times to initial ED assessment (< 10 min) and faster CT imaging (< 45 min) compared with non‐pathway care (> 50 and > 130 min) across EDs statewide.

**Conclusion:**

Use of the suspected acute stroke pathway appeared to vary across the four EDs, suggesting differences in pathway use and integration into everyday clinical workflows. Pathway use was associated with higher adherence to evidence‐based guidelines and improved timeliness of care. However, these findings are subject to confounding by other factors and should therefore be interpreted with caution.

## 1 Introduction

Globally, the World Health Organisation [1] estimates that over 62 million people are living with the long‐term effects of stroke. Over 440,000 Australians live with stroke's lasting effects, with one stroke occurring every 11 min [2]. Many of these individuals experience significant physical and mental disabilities, placing a substantial emotional, social, and financial burden on families and communities [3]. Given the widespread and life‐altering impact of stroke, early diagnosis and treatment are closely linked to improved outcomes and reduced disability [4]. Although the implementation of a clinical pathway has been shown to improve patient flow to specialised stroke wards, delays in communication, misdiagnoses and slow diagnostic processes can significantly worsen patient outcomes [4, 5]. In Australian emergency departments (EDs), stroke assessment is guided by national clinical standards and tools designed to support prompt and effective care [6]. These include early detection protocols, neurological assessments, imaging and diagnostics, and evaluation for eligibility for thrombolysis or endovascular therapy. To support this, the Australian Commission on Safety and Quality in Health Care, in collaboration with the Stroke Foundation, emphasises rapid assessment and management of suspected stroke cases [6]. Best practice guidelines recommend the use of validated screening tools and computed tomography (CT) imaging within 60 min of hospital arrival to differentiate between ischaemic and haemorrhagic strokes [5].

Ischaemic stroke, the most common type, is a major contributor to mortality and long‐term disability [7]. Reperfusion therapy via thrombolysis or mechanical thrombectomy is considered the gold standard for treatment [8]. Thrombolysis is ideally administered within 4.5 h of symptom onset, while mechanical clot retrieval may be performed up to 24 h later [2]. In contrast, haemorrhagic stroke, caused by bleeding in or around the brain, typically requires urgent neurosurgical intervention [9]. In this context, clinical pathways have emerged as a valuable tool for improving stroke care in EDs [10, 11]. These structured, evidence‐based sequences of investigation, diagnosis, and treatment help standardise care and reduce variability [12]. Early treatment has been shown to reduce death and long‐term disability and prevent complications; therefore, prompt assessment in the ED is essential for all suspected stroke presentations to optimise patient outcomes [3, 4]. In the fast‐paced ED environment, clinical pathways have been shown to reduce time to investigation and treatment, reduce ED length of stay (LoS) and align clinical decisions with evidence‐based best practices [13]. This is especially critical in stroke care, where the phrase ‘time is brain’ underscores the urgency of intervention [14]. Australia still lags behind similar nations in delivering timely stroke reperfusion therapy [15].

While several studies have examined clinical pathway development and implementation processes, the actual frequency and consistency of pathway use in routine practice remains poorly understood, particularly in ED settings [10]. There is limited evidence on how suspected acute stroke pathways are used across regional EDs, the consistency with which time‐critical assessment components are completed, and the extent of variation between EDs. Addressing these gaps is necessary to identify actionable targets for improvement and reduce unwarranted variation in acute stroke care delivery. This study's primary aim was to retrospectively assess the extent of suspected acute stroke pathway use across Tasmanian EDs. The secondary aims were [1] to assess adherence to evidence‐based guidelines as evidenced through completion of elements on the pathway [2] to evaluate waiting‐time metrics associated with pathway use; and [3] to assess whether there is variation in how the pathway is used between EDs.

This study forms part of a wider initiative, Implementing Pathways for Acute Care in Tasmania (IMPACT) that aims to promote adherence of pathways into current patient‐centred models of care across Tasmanian EDs [16].

## 2 Materials and Methods

### 2.1 Study Design

This retrospective observational study extracted data from digital medical records to identify eligible ED presentations for suspected stroke for the period 01 January 2021 to 31st December 2021.

### 2.2 Setting and Population

Under the Australian Standard Geographic Classification Remoteness Areas system, Tasmania is classified as predominantly regional, with substantial rural and remote areas [17]. Tasmania is Australia's only island state and is characterised by a high burden of chronic disease, lifestyle‐related risk factors, geographically dispersed communities and an ageing population [18]. The statewide suspected stroke pathway was implemented to address quality and safety concerns in acute stroke care. Initially trialled between June and August 2020, the paper‐based pathway was subsequently adopted across the three regions in Tasmanian EDs. Tasmania operates a hybrid medical record system in which both paper and electronic records are used during patient care. At discharge, all documents generated during the admission are scanned into the patient's digital record, providing a comprehensive combined source of coded, electronic and scanned paper information. The sample comprised four public EDs across the three regions of the Tasmanian Health Service as defined in the broader study protocol [16] and are summarised in Table 1 below. The Modified Monash Model (MM) classifies locations by remoteness and population from MM1 to MM7, using Australian Statistical Geography Standard—Remoteness Areas (ASGS‐RA) geography to guide equitable health workforce distribution across metropolitan, rural and remote Australia [17].

**TABLE 1:** Description of included public hospitals.

Hospital	Modified Monash (MM) ratings [17]	Annual ED presentations 2020–21 [19]	Services/capacity data source: [19, 20]
H1	MM 1—Metropolitan	71,960	Largest referral hospital (~501 beds). Statewide specialist emergency care with integrated neurology and stroke pathways, statewide neurosurgery onsite, and comprehensive tertiary services, for example, ICU/HDU
H2	MM 2—Regional Centre	44,200	~401 beds Primary Stroke Centre accreditation; onsite neurology with ED neurological assessment; no onsite neurosurgical surgery.
H3	MM 3—Large Rural Town	28,850	~160 beds. Acute stroke care with ED, imaging and general medical services; neurology delivered via statewide clinics; no onsite neurosurgery.
H4	MM 3—Large Rural Town	24,797	**~**95 beds. Essential regional ED with limited specialty capacity; stabilise‐and‐transfer model for stroke/neurological emergencies; no onsite neurosurgery.

### 2.3 Selection of Study Sample

The IMPACT protocol [16] provides a standardised framework for extracting and defining variables across Tasmanian EDs using a data dictionary (Supporting Information S1 EMA_IMPACT_DataDictionary.csv). The data applied standardised REDCap‐based procedures across the four EDs. The target sample of approximately 200 records across the three regions for suspected stroke (distributed 100 (H1): 50 (H2): 50 (H3/4)) was guided by the protocol document [16]. Using routinely collected ED admissions data, we assessed the representativeness of eligible cases based on diagnostic coding. Two independent research assistants extracted data from de‐identified digital medical records using explicit variable definitions from the published IMPACT data dictionary, with oversight from the broader research team to ensure consistency and data quality. The IMPACT methodology and data set has been previously published for fractured neck of femur [21].

### 2.4 Inclusion Criteria for Suspected Acute Stroke Clinical Pathway

Inclusion criteria were ED presentations with suspected stroke, identified by a primary International Classification of Diseases, 10th Revision (ICD‐10) diagnosis code and evidence of a CT brain scan. The ICD‐10 coding system is a globally used diagnostic tool developed by the World Health Organisation [22] to provide a standardised system for classifying diseases and health conditions. The primary diagnosis codes used in this study included conditions that may present as suspected stroke.

Primary diagnosis codes were grouped into 5 categories.

1. Ischaemic stroke (cerebral infarction)—code I63
2. Haemorrhagic stroke—codes I60‐I62
3. Unspecified stroke—code I64
4. Transient ischemic attack (TIA)—code G45
5. Other which included Bell's palsy G51.0, Viral disease B33.8, Dizziness/Vertigo R42, Syncope or collapse R55, Confusion R41, Migraine G43.9, Benign paroxysmal vertigo (BPPV) H81.1, Visual disturbance/impairment H53.9, Speech disturbance R47.8, Gait or mobility abnormalities R26.8, Malignant neoplasm of brain C71.9.

Only presentations with a CT scan were included, as non‐contrast CT (NCCT) is the essential first line imaging modality in the evaluation of suspected acute stroke and is universally performed to distinguish early between ischaemic and haemorrhagic stroke. NCCT was completed for all included patients in accordance with standard acute stroke protocols. Whilst NCCT serves as the foundational imaging step, it does not eliminate the need for additional warranted investigations such as CT angiography (CTA), CT perfusion (CTP), or MRI when clinically indicated. These further imaging studies were not used as inclusion criteria.

## 3 Data Collection

All suspected stroke presentations for 2021 that met the inclusion criteria were assigned a random number in Excel using the RAND() function before the target sample was selected. To allow for potential exclusions and missing data, we included a small number of additional presentations as contingency records. Coded extracts provided key administrative and clinical information for each presentation, which included acuity information, ED arrival time and time stamps for major clinical events. Triage nurses in Australia apply the Australasian Triage Scale (ATS) to prioritise patients [23]. Triage nurses undertake a nationally endorsed emergency triage education kit (ETEK) framework to ensure consistency for diverse ED presentations [24]. Demographic variables included sex and age. Pathway utilisation was identified through the presence of a scanned stroke pathway document, coded as completed if pathway element and key waiting time metrics were documented (Table 2); blank or absent forms were coded as non‐use unless verified via chart review.

**TABLE 2:** Selected suspected stroke pathway elements.

Early stroke identification	Initial observation and management	Waiting time metrics
Triage category (number) Code stroke (Y/N) ROSIER scale (Y/N) ECG (Y/N)	GoC (Y/N) Swallowing (Y/N) NIHSS (Y/N)	ED time to be seen (minutes) Door to CT (minutes) Door to treatment time (thrombolysis or endovascular therapy) (minutes) ED LoS (hours) Door to code stroke (minutes)

Pathway use variables utilised in data collection included binary indicators for completion of key elements as per evidence‐based guideline on the pathway document. These included code stroke activation, Recognition of Stroke in the Emergency Room (ROSIER) scoring, Goals of Care (GoC) documentation, National Institute of Health Stroke Scale (NIHSS) assessment, swallowing screening and ECG completion. Code stroke activation is a rapid hospital‐wide alert triggered when a patient shows sudden neurological deficits suggestive of stroke, enabling immediate mobilisation of the stroke team and urgent imaging before diagnostic confirmation based solely on clinical suspicion [25]. Within the pathway, ECG, ROSIER, and NIHSS collectively contribute to structured neurological assessment and early diagnostic accuracy [6]. Validated swallow assessment is recommended before oral intake to mitigate aspiration risk and GoC documentation reflects national standards promoting early clarification of treatment appropriateness and direction [6]. These elements align with Stroke Foundation guidance, which emphasises rapid, evidence‐based assessment processes to support timely imaging, escalation and reperfusion decision‐making [6].

Waiting time metrics included ED Time to be seen, door to CT, door to treatment intervals, time to code stroke activation and ED length of stay (LoS). Paper charts, scanned into the digital medical record, were reviewed when additional verification was required to ensure data completeness and accuracy. These sources included handwritten clinical notes, observation charts and pathway checklists. Paper records were consulted when digital entries were incomplete, ambiguous, or missing for key variables. Any inconsistencies between paper and electronic records were resolved collaboratively by the research team to ensure accurate transcription and consistent coding across all data elements.

## 4 Data Analysis

Results were imported and analysed in R studio (RStudio 2024.09.1+394). Continuous variables (age, waiting times and ED LoS) were summarised by mean whilst categorical variables were summarised by frequency and percentage.

### 4.1 Categorical Data

For categorical outcomes, we used chi square tests where counts and variability were sufficient and Fisher's exact test for small or skewed cells. To mitigate family wise error from multiple comparisons, we applied a Bonferroni correction to the 0.05 significance level; *p* < 0.0025 was considered statistically significant based on 20 statistical tests. We reported risk differences to quantify absolute changes between pathway and non‐pathway groups and relative differences to indicate proportional changes.

### 4.2 Continuous Data

For continuous outcomes, we used Welch's *t*‐test to compare means between independent groups because it is robust to unequal variances and sample sizes. After Bonferroni correction, *p* < 0.0025 denoted statistical significance.

## 5 Results

### 5.1 Participant Characteristics

Two hundred and seven presentations were identified for the study. Two presentations were excluded from the final analysis because their CT brain imaging was completed after they left the ED, leaving 205 presentations for analysis in the study. Table 3 provides a summary of the overall baseline participant characteristics for suspected stroke presentations. The mean age of the participants was 70.5 years, ranging from 18 to 97 years. The predominant age range was 80–89 years, with 53 (26%) of the presentations. Just over half the presentations were male (56%). Approximately double the proportion of presentations were triaged as category 2 when the pathway was used versus not used (83% vs. 41%). Unspecified Stroke (Stroke ICD‐10 Code I64) was the most frequently assigned primary diagnosis, with 64 (31%) presentations, followed by Ischemic Stroke/Cerebral Infarction (Stroke ICD‐10 Code I63) with 54 (26%) presentations.

**TABLE 3:** Overall baseline participant characteristics for stroke presentations.

	Overall study sample	Pathway used	Pathway not used
Presentations	205 (100%)	80 (39%)	125 (61%)
Mean age (years)	70.5	70.6	70.4
	*N* (%) = proportion of the total study sample presentations	*N* (%) = proportion of the study sample that used the pathway	*N* (%) = proportion of the study sample that did not use pathway
Age group			
49 and under	26 (13%)	11 (14%)	15 (12%)
50–59	20 (10%)	10 (13%)	10 (8%)
60–69	39 (19%)	11 (14%)	28 (22%)
70–79	50 (24%)	19 (24%)	31 (25%)
80–89	53 (26%)	24 (30%)	29 (23%)
90+	17 (8%)	5 (6%)	12 (10%)
Gender			
Male (%)	115 (56%)	36 (45%)	79 (63%)
Hospital			
H1	102 (50%)	28 (35%)	74 (59%)
H2	51 (25%)	25 (31%)	26 (21%)
H3	39 (19%)	24 (30%)	15 (12%)
H4	13 (6%)	3 (4%)	10 (8%)
Triage category			
Cat 1	7 (3%)	5 (6%)	2 (2%)
Cat 2	117 (57%)	66 (83%)	51 (41%)
Cat 3	70 (34%)	9 (11%)	61 (49%)
Cat 4	11 (5%)	0 (0%)	11 (9%)
Primary diagnosis			
Ischemic stroke (cerebral infarction) · I63	54 (26%)	27 (34%)	27 (22%)
Haemorrhagic stroke · I60·I62	10 (5%)	5 (6%)	5 (4%)
Unspecified stroke · I64	64 (31%)	28 (35%)	36 (29%)
Transient ischemic attack (TIA) · G45	30 (15%)	10 (13%)	20 (16%)
Other	47 (23%)	10 (13%)	37 (30%)

### 5.2 Use of Suspected Acute Stroke Pathway

To determine the extent of suspected acute stroke pathway use across the four ED departments, we compared usage when the pathway (document) was used and when it was not as summarised in Table 4. H3 had the highest proportion of pathway use (62%), followed by H2 (49%). H1 had the most ED presentations but a lower proportion of pathway document use (27%). H4 had the fewest presentations and lowest pathway use (23%).

**TABLE 4:** Proportion of presentations on pathway.

ED dept (presentations)	Pathway used *n* (%)	No pathway used *n* (%)
H1 (102 presentations)	28 (27%)	74 (73%)
H2 (51 presentations)	25 (49%)	26 (51%)
H3 (39 presentations)	24 (62%)	15 (38%)
H4 (13 presentations)	3 (23%)	10 (77%)

### 5.3 Adherence to Evidence‐Based Guidelines

To compare adherence to evidence‐based guidelines, we analysed completion rates of key clinical elements across all four ED when the pathway (document) was used and when it was not. This is summarised in Table 5. Pathway use was significantly associated with increased completion rates for several key clinical elements including ROSIER scale (80% vs. 11%) and NIHSS (70% vs. 12%). Code stroke activation was also significantly associated with pathway use (38% vs. 0%). Moderate gains were observed in Goals of Care (78% vs. 59%) and ECG (91% vs. 86%), while swallowing assessments remained unchanged. The absolute and relative difference for ROSIER scale (absolute 68.8%, 95% CI: 58.4–79.2; relative 614.3%, 95% CI: 331.0%–1084.0%) and NIHSS (absolute 58.0%, 95% CI: 46.5–69.5; relative 483.3%, 95% CI: 255.0%–858.0%) were statistically significant (*p* < 0.0025). Code stroke activation (absolute 37.5%, 95% CI: 26.9–48.1) was also statistically significant (*p* < 0.0025). However, a relative difference could not be calculated due to zero activation in the non‐pathway group. GoC documentation showed moderate improvements but did not meet the significance threshold. No meaningful differences were observed for ECG or Swallowing Screening, which had high completion rates in both groups. Thrombolysis occurred in 11% of pathway cases versus 1% without the pathway (absolute difference 10.5%, 95% CI: 3.4%–17.5%; *p* = 0.0011). Endovascular Therapy occurred in 5% versus 0% (absolute difference 5.0%, 95% CI: 0.2%–9.8%; *p* = 0.0221).

**TABLE 5:** Pathway element completion.

	Pathway used, *n* = 80 (%)	Pathway not used (*n* = 125)	Absolute difference % (95% CI)	Relative difference % (95% CI)	*p*
ROSIER scale^a^	64 (80%)	14 (11%)	68.8% (58.4 to 79.2)	614.3% (331.0% to 1084.0%)	< 0.0025
GoC	62 (78%)	74 (59%)	18.3% (5.7 to 30.9)	30.9% (9.0% to 58.0%)	0.0097
ECG	73 (91%)	108 (86%)	4.8% (−3.8 to 13.5)	5.6% (−4.0% to 16.0%)	0.3751
Swallowing	69 (86%)	108 (86%)	−0.1% (−9.8 to 9.5)	−0.2% (−11.0% to 12.0%)	1.0000
NIHSS^a^	56 (70%)	15 (12%)	58.0% (46.5 to 69.5)	483.3% (255.0% to 858.0%)	< 0.0025
Code stroke^a^	30 (38%)	0 (0%)	37.5% (26.9 to 48.1)	N/A*	< 0.0025
Thrombolysis	9 (11%)	1 (1%)	10.5% (3.4 to 17.5)	1306.2% (82.0% to 10,790.0%)	0.0011
Endovascular therapy	4 (5%)	0 (0%)	5.0% (0.2 to 9.8)	N/A*	0.0221

Abbreviation: N/A, not applicable, cannot be calculated.

^a^ Statistical significance *p* < 0.0025.

### 5.4 Waiting‐Time Metrics

To evaluate waiting‐time metrics associated with pathway use, we compared waiting times when the pathway was used and when it was not across the EDs. The waiting time metrics are summarised in Table 6. Use of the suspected stroke pathway was associated with reduced waiting times for ED Time to be seen and CT brain imaging (*p* < 0.0025). Patients on the pathway waited less than 10 min to be seen and under 45 min for CT brain imaging, compared to over 50 and 130 min respectively when the pathway document was not used. No significant difference was found in ED LoS. Thrombolysis wait data had only seven valid entries, with a mean of 123 min and a wide range from 79 to 205 min. The Code Stroke wait time data included 20 entries, with a mean of −8.9 min and a range from −149 to 78 min. The negative values represent pre‐notification, where stroke teams were alerted before the patient arrived.

**TABLE 6:** Comparison of waiting time metrics.

Metric	Pathway used, *n* = 80 mean (SD)	Pathway not used, *n* = 125 mean (SD)	*p*
Code stroke wait (min)	−8.90 (SD 39.74)	None recorded	—
ED time to be seen (min)^a^	9.73 (SD 11.19)	53.71 (SD 77.65)	< 0.0025
CT brain imaging wait (min)^a^	42.55 (SD 55.01)	130.82 (SD 125.43)	< 0.0025
Thrombolysis wait (min)	79.00 (SD 43.17)	None recorded	—
ED LoS (hours)	11.68 (SD 13.86)	13.49 (SD 11.25)	0.329

^a^ Statistical significance *p* value < 0.0025.

### 5.5 Variation Between EDs

To assess whether there is any variation in how the pathway is used between EDs, we compared adherence to evidence‐based guidelines (element completion when the pathway was used) (Table 7). When the pathway was used, element completion rates were generally high and consistent across sites, and no differences reached the Bonferroni significance threshold (*p* < 0.0025). Comparisons involving H4 (*n* = 3) were uniformly non‐significant and should be interpreted descriptively only due to very low statistical power.

**TABLE 7:** Comparison of element completion between hospital EDs when pathway is used.

Element	H1, *n* = 28 (%)	H2, *n* = 25 (%)	H3, *n* = 24 (%)	H4, *n* = 3 (%)	*p*					
					H1/H2	H1/H3	H1/H4	H2/H3	H2/H4	H3/H4
ROSIER	19 (68%)	23 (92%)	20 (83%)	2 (67%)	0.043	0.336	1.000	0.417	0.298	0.474
GoC	20 (71%)	22 (88%)	17 (71%)	3 (100%)	0.183	1.000	0.544	0.171	1.000	0.545
ECG	23 (82%)	24 (96%)	23 (96%)	3 (100%)	0.196	0.199	0.566	1.000	1.000	1.000
Swallowing	21 (75%)	21 (84%)	24 (100%)	3 (100%)	0.509	0.011	0.544	0.110	1.000	1.000
NIHSS	19 (68%)	16 (64%)	20 (83%)	1 (33%)	0.780	0.336	0.282	0.196	0.543	0.115
Code stroke	8 (29%)	11 (44%)	11 (55%)	0 (0%)	0.267	0.253	1.000	1.000	0.258	0.248
Thrombolysis	4 (14%)	3 (12%)	2 (10%)	0 (0%)	1.000	0.674	1.000	1.000	1.000	1.000

## 6 Discussion

This study sought to assess the extent of suspected acute stroke pathway use, adherence to evidence‐based guidelines as evidenced through completion of elements on the pathway, waiting‐time metrics, and pathway use variation across regional EDs in Tasmania.

In contrast to prior evidence suggesting lower and more variable adherence to organised stroke processes in rural hospitals compared with metropolitan centres [26], our study did not identify differences in adherence to evidence‐based guidelines across sites when the pathway was used. The observed relationship between pathway use and more timely imaging aligns with rural stroke literature, where early diagnostic steps are often achieved efficiently, while delays more commonly arise during post‐imaging decision‐making and inter‐hospital transfer processes [27]. Interpretation of these findings should consider the potential influence of confounding and contextual factors which may independently affect imaging timeliness and ED outcomes. Additionally, ED LoS was shorter at the rural town site compared with the regional centre. Prior studies suggest that ED LoS in rural and regional hospitals is largely shaped by system‐level constraints, including bed availability, transfer logistics, and access to specialist services, rather than pathway use in isolation [28].

Stroke pathway utilisation in our study appeared to vary between hospitals (27%–62%), suggesting a persistent challenge in achieving consistent stroke care delivery across Australia [29, 30]. This variation aligns with national evidence demonstrating that, despite established clinical standards, a national stroke registry, and designated Centres of Excellence, Australia continues to lag behind comparable high‐income countries in the timeliness of care [29, 30]. In 2022, only 27% of patients received reperfusion therapy within one hour of hospital arrival, compared with 82% in Sweden, 75% in the United States and 61% in the United Kingdom [15]. Whilst incremental improvements have been observed in selected care processes such as swallow screening, discharge planning, and antihypertensive prescribing at discharge, delays and wide inter‐hospital variation in reperfusion timeliness persist in Australia [26, 31].

A central suggestion in our cohort is under‐recognition at triage. Only 57% of suspected stroke presentations were assigned ATS 2, despite policy that all suspected strokes should be triaged as Category 2 [15]. This has cascading effects. Patients triaged ATS 2 were far more likely to be placed on the pathway than those assigned other categories (83% vs. 41%), indicating that triage acuity, not the pathway itself, may be an important determinant of downstream workflow timeliness. Inadequate recognition at triage may delay clinical assessment, imaging priority, and pathway use, culminating in longer ED LoS. Viewed through this lens, our site differences (e.g., shorter CT waits at H3) could be attributed to recognition rather than pathway use alone. This interpretation is consistent with literature showing that post‐imaging processes tend to be the principal source of downstream delays [27, 30, 32]. While national compliance metrics remain important, our data suggest that improving the sensitivity of stroke recognition at triage (ensuring ATS 2 for all suspected cases) is a potential key, modifiable system lever. Notably, similar patterns of ED‐to‐CT and in‐hospital delay linked to early process gaps have been described in tertiary settings [33], reinforcing the need to prioritise accurate triage categorisation as the first step in time‐critical stroke care.

Code stroke activation referenced in this suspected acute stroke pathway reflects a protocol‐driven process and does not operate with the same level of coordination or automation typically associated with a code STEMI response. The operational definition has evolved since the study period. In our study, code stroke activations and thrombolysis administrations occurred exclusively among patients managed on the suspected stroke pathway. This does not imply that pathway use was a prerequisite for treatment but may reflect how the pathway was operationalised in practice, with clinicians more likely to initiate escalation processes for patients formally placed on the pathway. International evidence provides some suggestions why code stroke activation is often low as our study reported. Code stroke is generally reserved for hyperacute reperfusion candidates, whereas broader pathway elements are applied to a wider suspected stroke population, including TIA and stroke mimics [34]. In our study, the other group, comprising conditions that mimic stroke, accounted for 23% of presentations. This proportion is consistent with existing evidence indicating that stroke mimics constitute approximately 20%–40% of suspected ED stroke presentations, thereby reducing the proportion of patients eligible for code stroke activation [34]. Screening tools such as ROSIER have high sensitivity but moderate specificity, supporting early case‐finding but limiting suitability as a standalone trigger [35]. Variability and non‐compliance in activation practices across EDs further contribute to selective activation [25].

Beyond documenting adherence to evidence‐based guidelines, waiting time metrics and variability in pathway use, the study provides practice level data on how stroke pathways are used within everyday clinical workflows. The issues identified in this study may not be unique to Tasmania, and it is possible that similar challenges occur more broadly. Consequently, the lessons generated here could potentially be applicable to other comparable health systems that face difficulties in using pathways in everyday clinical practice.

## 7 Study Limitations

This retrospective study is subject to several limitations. Retrospective designs cannot fully control for confounding variables such as staffing levels, illness severity, or comorbidities, all of which may influence completion of care elements and delays in imaging or treatment. Reliance on existing clinical records introduces potential inaccuracies, as documentation may be incomplete, inconsistent, or erroneous. Although electronic health records hold considerable promise, substantial clinical information remains embedded within unstructured free text. Manual chart abstraction is therefore vulnerable to missing data, conflicting entries, ambiguous or illegible notes, inconsistent coding, and transcription errors, all of which may compromise data quality and scientific validity [36].

This study assessed a suspected acute stroke pathway that is initiated prior to definitive diagnostic classification. Consequently, the cohort included ischaemic stroke, haemorrhagic stroke, TIA and stroke mimics, which differ in urgency, imaging requirements, treatment eligibility, specialist involvement and transfer needs. These differences suggest that pathway elements (e.g., code stroke activation, ROSIER, NIHSS, swallowing screen and time‐critical imaging) may not be equally relevant, feasible, or beneficial across subtypes. Aggregating subtypes in the analysis may therefore obscure subtype specific patterns or dilute true effects [37]. Moreover, site level variation in the handling of suspected haemorrhage or mimics (e.g., rapid neurosurgical transfer versus local management) could differentially affect waiting time metrics. Accordingly, findings should be interpreted with caution: observed associations reflect performance within a suspected stroke workflow and may not generalise uniformly to any single confirmed subtype.

This study relied on unadjusted comparisons; therefore, observed associations may be influenced by patient, service and temporal factors (e.g., age, comorbidity, illness severity proxies and site level resources). Without comprehensive multivariable adjustment, estimates may reflect residual confounding rather than causal effects [38]. We used Fisher's exact test for small cells to ensure valid *p* values and applied Bonferroni correction to limit Type I error; however, this approach increases the risk of Type II error in a modest sample. Multiple comparisons also heighten the likelihood of Type I error; thus, statistically significant findings, particularly from subgroup analyses, should be viewed as exploratory.

## 8 Recommendations and Future Directions

While the quantitative analyses highlight systemic contributors to delays in acute stroke care, including pre‐hospital recognition challenges, suboptimal triage categorisation, imaging bottlenecks, and variability in clinical assessment, the mechanisms underlying these patterns are best clarified through qualitative enquiry. As part of the broader research program, a follow‐up qualitative study is planned to explore how local workflows, staffing dynamics, physical layout, competing clinical priorities, escalation pathways and informal workarounds shape real‐world practice. The qualitative insights are likely to shed light on operational, cultural, and contextual factors that are not captured in routine metrics but are critical for achieving sustainable improvements.

## 9 Mitigation of Bias

We implemented comprehensive bias‐reduction strategies aligned with evidence [39]. Data were collected using a piloted, standardised instrument with predefined variables and a clear data dictionary. Two blinded, trained research assistants independently extracted data under academic supervision. Blinding to study hypotheses, regular calibration meetings, and consensus resolution of discrepancies ensured consistency. Chart reviews complemented digital records, and investigator roles were disclosed. Data were de‐identified prior to analysis. These measures enhanced transparency, minimised abstractor and selection bias and strengthened methodological rigour.

## 10 Conclusion

Use of the suspected acute stroke pathway appeared to vary across the four EDs, suggesting differences in pathway use and integration into everyday clinical workflows. Pathway use was associated with greater adherence to evidence‐based guidelines and timeliness of care. However, the results are confounded by other factors and should be interpreted with caution.

## Author Contributions

The authors confirm contribution to the paper as follows: Study conception and design: I.T.M., S.J.P., P.G., E.T., V.T. Data collection: I.T.M., S.J.P., P.G. Analysis and interpretation of results: I.T.M., S.J.P., P.G., E.T., V.T. Draft manuscript preparation: I.T.M., S.J.P., P.G., E.T., V.T. All authors reviewed the results and approved the final version of the manuscript.

## Funding

This work was supported by the National Health and Medical Research Council, MRF2018041.

## Ethics Statement

The study received Ethics approval from the University of Tasmania Human Research Ethics Committee (HREC)‐ Project ID: 28825 and site‐specific approvals from the three regions. A waiver of consent was sought and approved for use of medical record data.

## Conflicts of Interest

V.T. is a section editor for the EMA.

## Supporting information


**Data S1:** EMA_IMPACT_DataDictionary.

## Data Availability

The data that support the findings of this study are available from the corresponding author upon reasonable request.
